# Autoantibody with Cross-Reactivity between Insulin and Ductal Cells May Cause Diabetic Mastopathy: A Case Study

**DOI:** 10.1155/2012/569040

**Published:** 2012-04-22

**Authors:** Katsutoshi Miura, Chikako Teruya, Nasu Hatsuko, Hiroyuki Ogura

**Affiliations:** ^1^Departments of Health Science, Pathology and Anatomy, Hamamatsu University School of Medicine, 1-20-1 Handa-yama, Higashi-ku, Hamamatsu 431-3192, Japan; ^2^Division of Breast Surgery, Hamamatsu University School of Medicine, 1-20-1 Handa-yama, Higashi-ku, Hamamatsu 431-3192, Japan; ^3^Division of Radiology, Hamamatsu University School of Medicine, 1-20-1 Handa-yama, Higashi-ku, Hamamatsu 431-3192, Japan

## Abstract

Lymphocytic mastopathy or diabetic mastopathy is a benign breast disease characterized by dense fibrosis, lobular atrophy, and aggregates of lymphocytes in a periductal and perilobular distribution. The condition usually affects women with a long history of diabetes mellitus (DM) and also those with autoimmune disorders. While the pathogenesis is unknown, a particular type of class II human leukocyte antigen has been associated with this disease. Herein, we report a case of diabetic mastopathy which clinically and radiologically mimicked primary breast neoplasms. The patient was a 74-year-old woman with a 31-year history of DM type II who presented with multiple firm lumps in bilateral breasts. Findings from mammography, ultrasonography, and magnetic resonance imaging of the breasts revealed an abnormal appearance which suspiciously resembled malignancy. An aspiration cytology specimen showed atypical accumulation of lymphoid cells, leading us to suspect lymphoma. Histology of an excisional biopsy showed the characteristic appearance of lymphocytic mastopathy, which predominantly consisted of B-lymphocytes. Autoantibodies in her serum reacted positively against her ductal epithelium as well as other diabetic and nondiabetic breast ductal cells. An antigen absorption test with insulin revealed attenuating intensity according to insulin concentration. These anti-insulin antibodies produced in the DM patient may cause ductitis because of antigen cross-reactivity.

## 1. Introduction

Diabetic mastopathy, also known as lymphocytic mastitis or lymphocytic mastopathy, is occasionally seen in women who have longstanding diabetes mellitus (DM). The typical presentation is suspicious fibrous breast lumps. Pathology reveals dense keloid-like fibrosis and periductal, lobular, or perivascular lymphocytic infiltration [1, 2]. While the disease pathogenesis is unknown, the condition may be the result of an autoimmune reaction since the histological features are similar to those seen in other autoimmune diseases [3]. Here we present a case of diabetic mastopathy, which proposes the pathogenetic hypothesis that autoantibodies against insulin may cause autoimmune mastitis because of cross-reactivity to mammary ducts.

## 2. Case Study

A 74-year-old woman complained of a right breast mass which had appeared four years earlier. She had a 31-year history of DM with insulin treatment.

**Figure 1:**
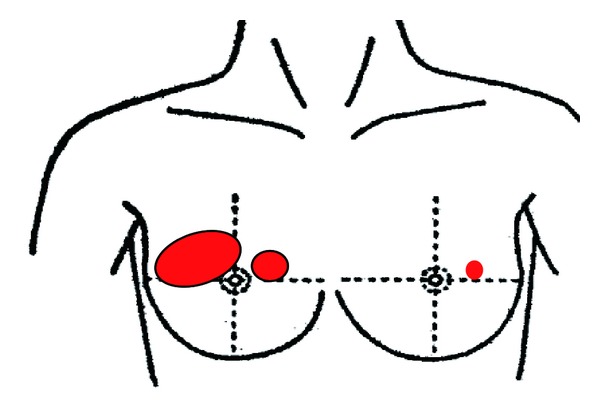
Three separate tumors of the breast by palpation and imaging, two hard tumors in the CDA and A areas of the right breast and one nonpalpable solid tumor in the C area of the left breast were found.

Upon palpation, two hard lumps in the right breast were discovered (Figure 1, right breast). One tumor had a maximum diameter of 45 mm with a clear margin over the CAE area, and the other was 25 mm in diameter with a clear margin in the A area 25 mm from the nipple. No growths were palpated in the left breast, and surface lymph node swelling was not present.

A mediolateral oblique and craniocaudal mammographic view of the right breast demonstrated a dense microlobulated mass 16 × 21 mm in size with a small round calcification in the MI area 43 mm from the nipple. Abnormality including carcinoma was suspected. The left breast showed no remarkable changes.

Ultrasonography revealed an ill-defined, irregular-shaped hypoechoic area with acoustic shadowing at the 9-to 12-o'clock position of the right breast. At the 2-o'clock position in A area 25 mm from the nipple, a well-defined hypoechoic mass with posterior acoustic shadowing was seen. At the 2.5-o'clock position 25 mm from the nipple of the left breast, an ill-defined, irregular-shaped hypoechoic area with acoustic shadowing was seen (Figure 1, left breast). These masses did not exclude the possibility of carcinoma.

**Figure 2:**
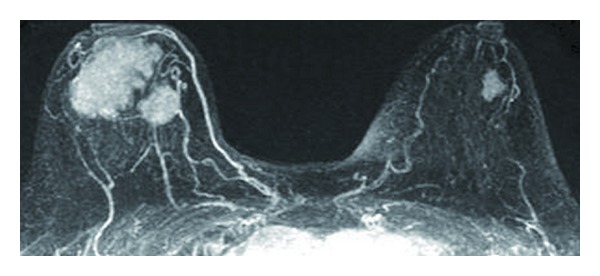
Magnetic resonance imaging of the breast dense microlobulated masses with irregular margins, 43 × 52 × 37 mm and 17 × 23 × 14 mm, were seen in the CDA and A areas of the right breast, respectively. In the C area of the left breast, a similar characteristic mass, 11 × 20 × 15 mm, was also seen.

Dense microlobulated masses with irregular margins, 43 × 52 × 37 mm and 17 × 23 × 14 mm, were revealed by magnetic resonance imaging (Figure 2), in the CDA and A areas of the right breast, respectively. In the C area of the left breast, a similar characteristic mass, 11 × 20 × 15 mm, was also seen.

A few clusters of ductal cells and many small lymphocytes were present in an aspiration cytology specimen, which suggested malignant lymphoma.

**Figure 3:**
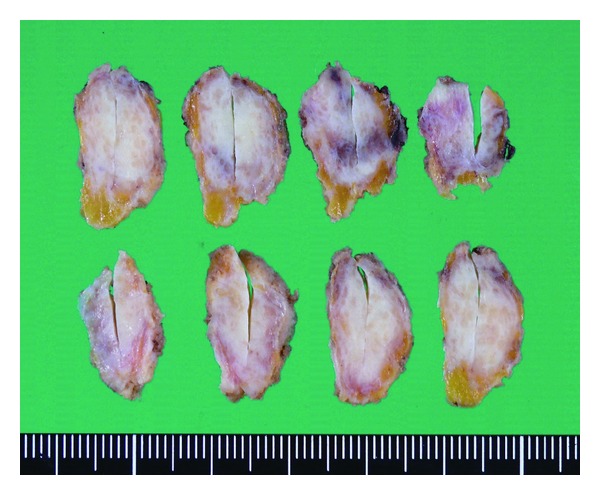
Macroscopic cut sections of the tumor A well-defined elastic hard mass, 2.5 × 2 × 1 cm, was excised from the A area of the right breast. Meshwork composed of whitish fibers separating atrophic fatty breast tissue was observed.

A well-defined elastic hard mass from the A area of the right breast was removed because of suspicion of malignancy. Cut sections revealed a meshwork composed of whitish fibers separating atrophic fatty breast tissue (Figure 3).

**Figure 4:**
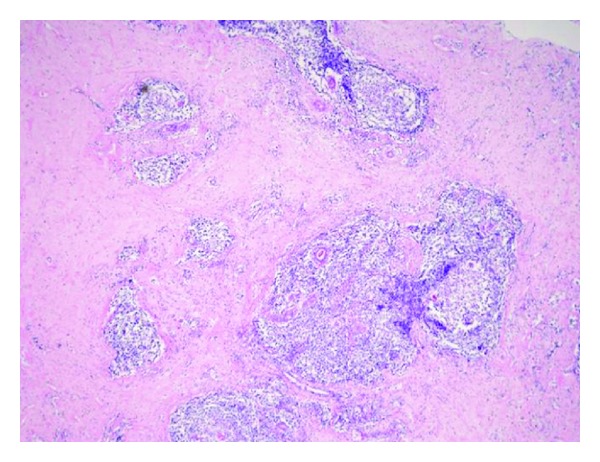
Histology of the breast tumor (×40). Many small lymphocytes infiltrated periducts with surrounding dense collagen fibers, accompanied by lymph follicles with germinal centers.

Histology revealed lymphocytic ductitis or lobulitis with surrounding dense fibrosis (Figure 4).

**Figure 5:**
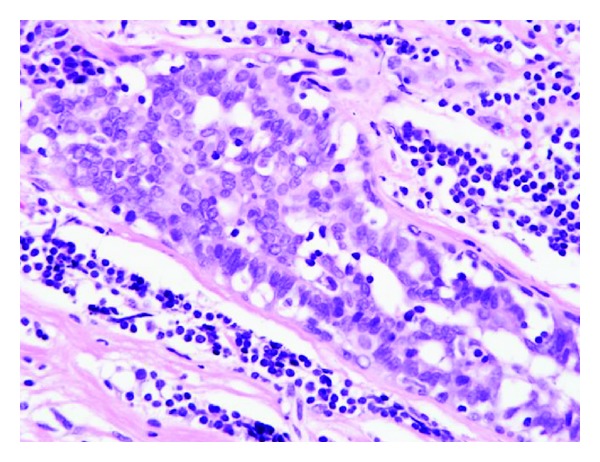
High magnification of the tumor (×400). Small lymphocytes infiltrated between ductal epithelia forming lymphoepithelial lesions.

**Figure 6:**
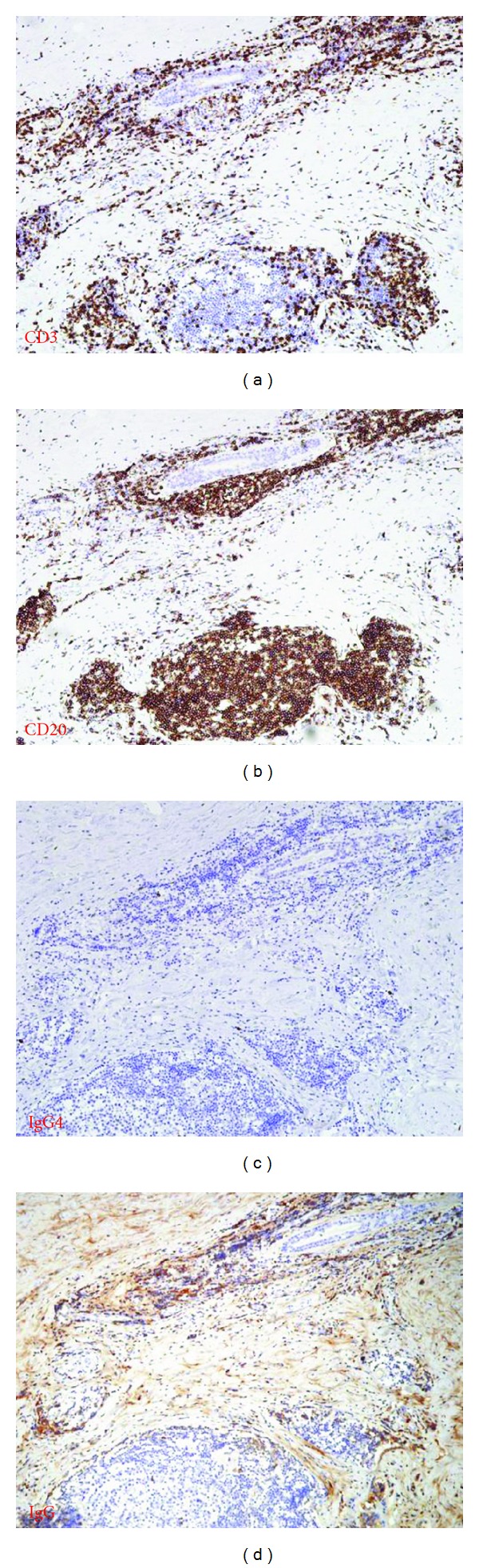
Lymphocyte markers by immunostaining. Infiltrating lymphocytes consisted more of CD20-positive cells than CD3-positive cells. IgG4-positive lymphocytes were rarely found compared with IgG positive cells.

Small lymphocytes had infiltrated between ductal epithelia forming lymphoepithelial lesions (Figure 5). These lymphocytes comprised more of CD20-positive cells (Figure 6) than sparse CD3-positive cells. IgG4-positive lymphocytes were rarely found compared with IgG-positive cells.

On investigating the pathogenesis of this disease, autoantibodies against breast tissues were detected by an indirect immunoperoxidase technique using the patient's serum as the primary antibody [4]. The endogenous peroxidase was inactivated by dipping sections in 0.3% hydrogen peroxide and 0.1% sodium azide for 10 min. The sections were then washed in phosphate buffered saline (PBS) three times and soaked with 30% normal rabbit serum in PBS for 10 min to avoid nonspecific absorption. The patient's serum was applied as the primary antibody at serial concentrations of 1 : 50, 1 : 100, 1 : 200, 1 : 400, and 1 : 800. Normal human serum was used as a negative staining control. Incubation was at 4°C or room temperature for 1.5 h. After washing three times in PBS, sections were incubated with anti-human IgG, IgM, IgA, kappa, and lambda antibodies labeled with horseradish peroxidase (DAKO, Kyoto, Japan) at a 1 : 50 dilution for 30 min. After washing three more times in PBS, sections were stained with diaminobenzidine-hydrogen peroxide solution.

The antigen absorption method was performed by mixing patient serum with human recombinant insulin (Invitrogen, Carlsbad, CA, USA) for 1 h at room temperature and 1.5 h at 4°C followed by centrifugation. The insulin concentration was increased stepwise from 40 ng/mL to 400 *μ*g/mL 10 times. Bovine albumin was used as a negative control.

**Figure 7:**
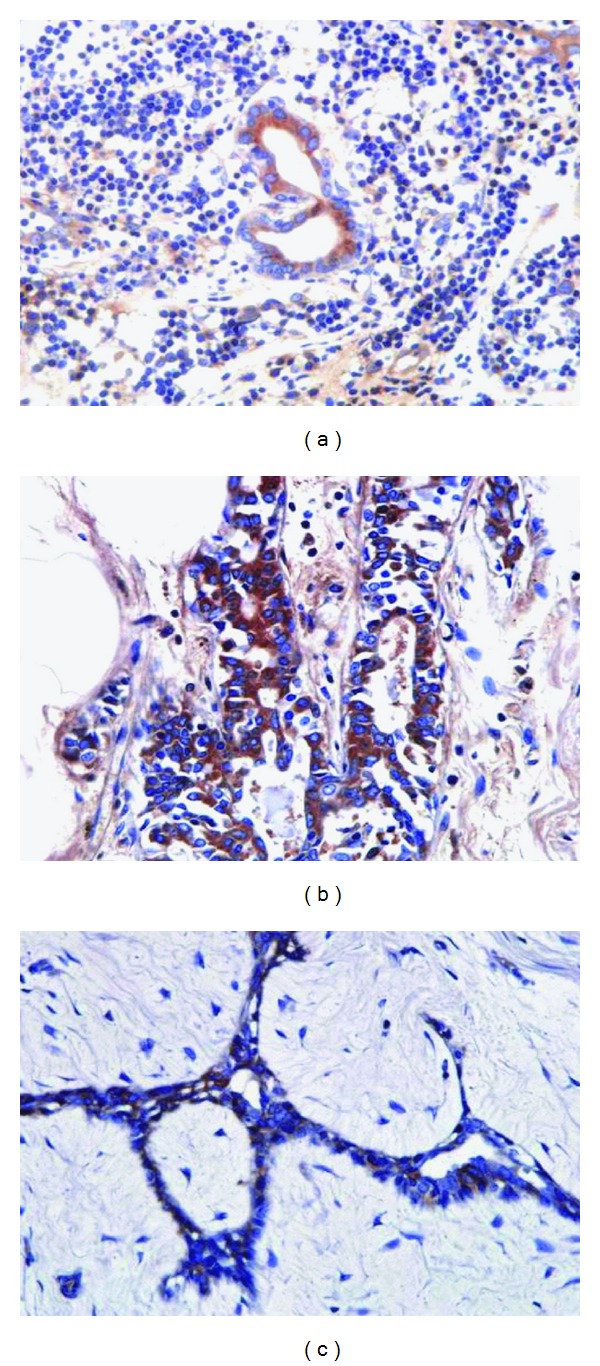
Autoantibody reacting to the patient's own (a), other DM patient's (b), and non-DM patient's (c) breast tissue. Using the patient's serum as the primary antibody, ductal epithelium showed positive staining at a 1 : 200 dilution.

Using the patient's serum as a primary antibody resulted in positive staining of ductal epithelia at dilutions of 1 : 100 to 1 : 200 (Figure 7(a)). Ductal epithelia from other diabetic (Figure 7(b)) or nondiabetic patients (Figure 7(c)) also showed a positive reaction.

**Figure 8:**
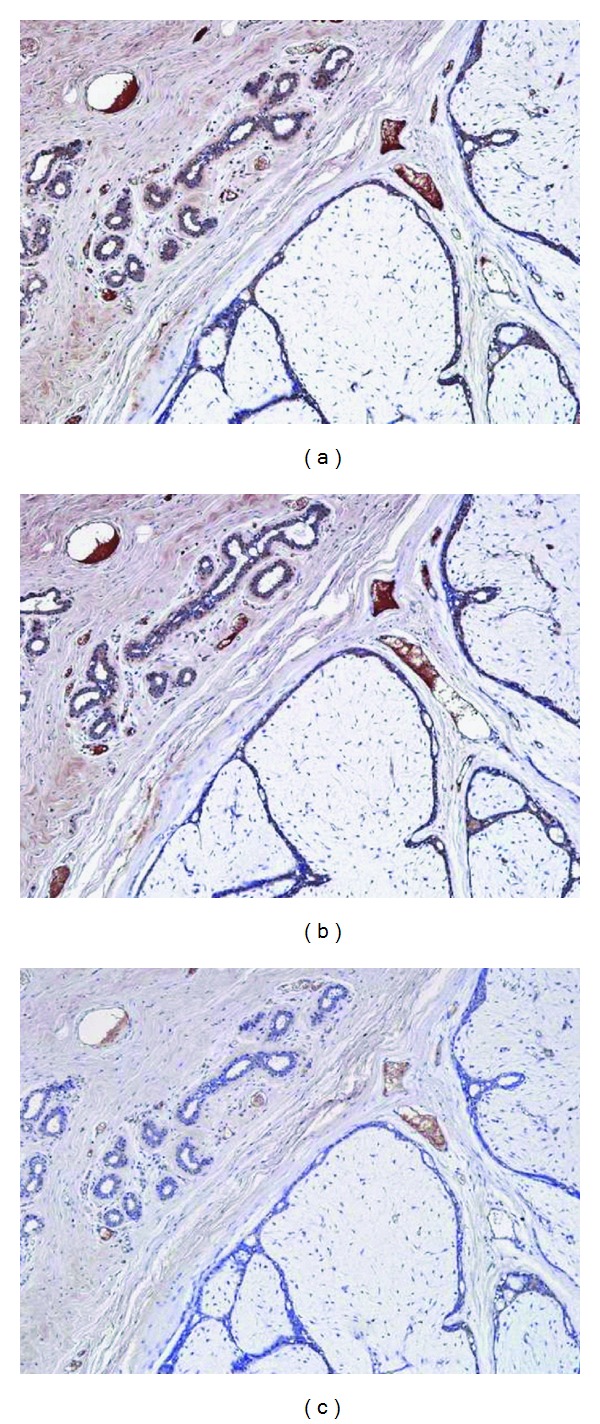
Antibody absorption test with insulin added to the primary serum at 40 ng/mL (a), 4 *μ*g/mL (b), and 40 *μ*g/mL (c). Staining intensity attenuated corresponding to insulin concentration. At 40 ng/mL, positive staining of ductal epithelia remained, while at 4 *μ*g/mL, staining intensity decreased significantly and almost disappeared at 40 *μ*g/mL.

The antibody absorption test with insulin showed attenuating staining intensity corresponding to increasing insulin concentrations. At 40 ng/mL, positive staining of ductal epithelia remained (Figure 8(a)), while at 4 *μ*g/mL, staining intensity decreased significantly (Figure 8(b)) and almost disappeared at 40 *μ*g/mL (Figure 8(c)).

## 3. Discussion

An association between DM and fibrous breast disease was reported initially in 1984 by Soler and Khardori [1] who described 12 patients with longstanding type 1 DM, multiple diabetic complications, and palpable breast masses. Three years later, the term diabetic mastopathy was coined for the characteristic combination of connective tissue overgrowth with perivascular lymphocytic infiltration of the lesion. As similar histopathological features were observed in patients with autoimmune disease, and increased human leukocyte antigen-DR expression by breast epithelial cells was observed, the hypothesis that this disease was a connective tissue disorder or an autoimmune disorder was raised [3]. B-cell-predominant lymphoid infiltrates forming lymphoepithelial lesions were present [5] as seen in this case; however, the B cells were polyclonal and the risk for lymphoma is low in diabetic mastopathy.

Lymphoplasmacytic infiltration with fibrosis reminded us of the recently proposed entity, IgG4-related sclerosing disease, which has been found in the whole body [6, 7]. Zen et al. reported an inflammatory pseudotumor of the breast associated with this sclerosing disease [7]. Since the present case showed little plasma cell infiltration and rare IgG4-positive plasma cells in the lesion, the possibility of IgG4-related sclerosing mastitis is very low.

The autoantibody against insulin and breast ductal cells indicates that there may be a connection to feeding cow milk. There are some reports which indicate that short-term exclusive breastfeeding or early exposure to cow milk formulas can increase the risk of type 1 DM [8, 9]. Cow milk contains bovine insulin [8] and possibly breast ductal cell components. Autoantibodies to insulin, GAD65, and protein tyrosine phosphatase-related IA-2 molecules were induced more in infants who had been exclusively breastfed for less than two months than those who had been exclusively breastfed for at least four months [9].

Molecular mimicry is characterized by an immune response to an environmental agent that cross-reacts with a host antigen, resulting in disease. This pathogenesis has been implicated in the pathogenesis of diabetes [10].

In conclusion, this is the first paper of a diabetic mastopathy patient having autoantibodies against insulin and ductal cells with cross-reactivity.
